# Assessing the Presence of Phosphoinositides on Autophagosomal Membrane in Yeast by Live Cell Imaging

**DOI:** 10.3390/microorganisms12071458

**Published:** 2024-07-18

**Authors:** Jing-Zhen Song, Yi-He Feng, Valentina Sergevnina, Jing Zhu, Hui Li, Zhiping Xie

**Affiliations:** State Key Laboratory of Microbial Metabolism, School of Life Sciences and Biotechnology, Shanghai Jiao Tong University, Shanghai 200240, China

**Keywords:** phosphoinositides, autophagy, protein trafficking, yeast

## Abstract

The formation of autophagosomes mediating the sequestration of cytoplasmic materials is the central step of autophagy. Several phosphoinositides, which are signaling molecules on the membrane, are involved in autophagy. However, it is not always clear whether these phosphoinositides act directly at the site of autophagosome formation, or indirectly via the regulation of other steps or pathways. To address this question, we used a set of phosphoinositide probes to systematically examine their potential presence on autophagosomal membranes in yeast (*Saccharomyces cerevisiae*). We verified the specificity of these probes using mutant cells deficient in the production of the corresponding phosphoinositides. We then examined starved yeast cells co-expressing a phosphoinositide probe together with an autophagosomal membrane marker, 2Katushka2S-Atg8. Our data revealed that PtdIns(4,5)P_2_ and PtdIns(3,5)P_2_ were mainly present on the plasma membrane and vacuolar membrane, respectively. We observed only occasional co-localization between the PtdIns(4)P probe and Atg8, some of which may represent the transient passage of a PtdIns(4)P-containing structure near the autophagosomal membrane. In contrast, substantial colocalization of the PtdIns(3)P probe with Atg8 was observed. Taken together, our data indicate that only PtdIns(3)P is present in a substantial amount on the autophagosomal membrane. For other phosphoinositides involved in autophagy, either their presence on the autophagosomal membrane is very transient, or they act on other cellular membranes to regulate autophagy.

## 1. Introduction

**Figure 1:**
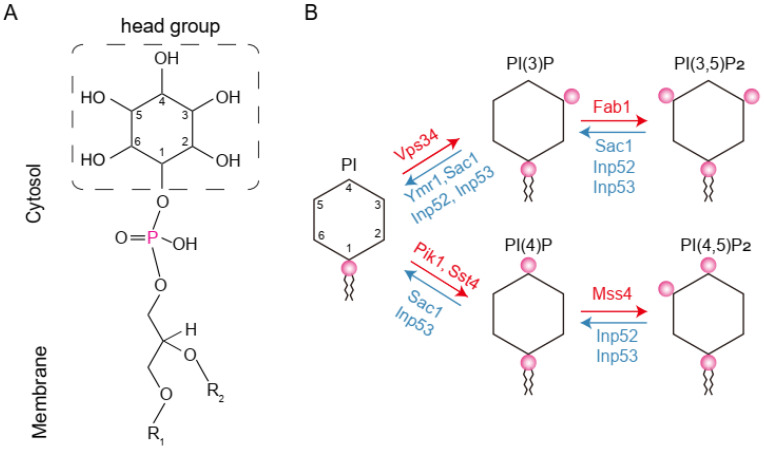
Structure and metabolism of phosphoinositides. (**A**) Diagram of PtdIns. PtdIns is a type of membrane phospholipid carrying an inositol ring as the head group. The hydroxyl groups at D3, D4, and D5 positions on the inositol ring can be phosphorylated to generate various derivative species, i.e., the phosphoinositides. (**B**) Enzymes involved in the interconversion of phosphoinositides in yeast. Red and blue arrows indicate the reactions mediated by kinases and phosphatases, respectively. Enzymes involved are marked in the same color scheme. Note that specificity of the phosphatases is low.

Phosphorylated derivatives of phosphatidylinositol (PtdIns), also known as phosphoinositides, are an important class of membrane lipids in eukaryotic cells. As signaling lipids, they play pivotal roles in various cellular functions such as cytoskeletal organization, ion channel regulation, and membrane trafficking [1,2,3]. Like many other glycerophospholipids, the head group of phosphoinositide, an inositol ring, faces the cytosol. The inositol ring can be reversibly phosphorylated by a network of conserved kinases and phosphatases on D3, D4, and or D5 positions to generate different phosphoinositides, including mono-, double-, or triple-phosphorylated derivatives (Figure 1) [4,5,6]. Dynamic regulation of their concentration and subcellular distribution is critical for maintaining cell homeostasis and responding to external stimuli [7,8].

The combinatorial phosphorylation of the inositol ring at different positions essentially generates a lipid “code” to regulate downstream processes. The phosphorylated inositol rings, present on the cytoplasmic leaflet of cellular membranes, execute their functions by recruiting specific effector proteins that possess certain phosphoinositide-binding domains to discriminate the distinct codes [9,10]. For instance, during the maturation of endosomes, sorting nexin proteins are recruited to endosomes to mediate protein sorting and membrane deformation. Sorting nexins are recruited via the phosphatidylinositol 3-phosphate (PtdIns(3)P) binding ability of their PX (*P*ho*X* homology) domains. The PX domain is among a group of commonly encountered domains in our proteome that can recognize phosphoinositides with different specificity and affinity, including PH (*P*leckstrin *H*omology), FYVE (*F*ab1, *Y*OTB, *V*ac1, and *E*EA1), and PROPPIN (β-*PROP*ellers that bind *P*hospho*IN*ositide*S*) domains. These domains discriminate different phosphoinositides by a combination of non-specific electrostatic interactions and stereoselective coordination of the phosphoinositides headgroups. In lipid signaling research, these domains are often used as probes to label phosphoinositides in live cells.

Autophagy is a critical cellular degradation process involving the de novo formation of autophagosomes to deliver cytoplasmic components to lysosomes or vacuoles. Autophagosomes are double-membrane vesicles. They are formed at distinct subcellular sites via the expansion and deformation of precursor membrane sacs. It is important that the activity of autophagy be controlled at an appropriate level. Both hyperactivity and hypoactivity of autophagy have been associated with human diseases, such as neurodegeneration, cancer, and metabolic diseases. Phosphoinositides are known to be key regulators in multiple stages of autophagy [11,12,13]. While PtdIns 3-phosphate (PtdIns(3)P) and the kinase that produces it, PtdIns 3-kinase (PtdIns(3)K), are extensively studied for their roles in autophagy across different organisms, the functions of other phosphoinositides in this process are less well understood [12,14,15]. PtdIns 4-kinases (PtdIns(4)Ks), which localize to different cellular compartments, appear to have distinct roles in autophagy regulation [16]. For instance, the Golgi-localized PtdIns(4)K influences autophagy initiation, whereas the plasma membrane-localized PtdIns(4)K is implicated in autophagosome–lysosome fusion. Double phosphorylated PtdIns also participate in autophagy regulation. PtdIns 3,5-diphosphates (PtdIns(3,5)P_2_) is suggested to negatively regulate autophagy [17,18]. The role of PtdIns 4,5-diphosphates (PtdIns(4,5)P_2_) in autophagy appears to be species-dependent [19,20,21,22,23].

To better understand how these phosphoinositides regulate autophagosome formation, it is crucial to determine the subcellular localization of phosphoinositides under autophagy-inducing conditions. In particular, whether or not a particular phosphoinositide is present at the site of autophagosome formation provides an indication of whether the regulation is direct or indirect. Although many studies focusing on particular phosphoinositides have examined their subcellular localization individually, a systematic comparison among phosphoinositides under the same condition has not been performed, complicating the interpretation of existing data [24,25,26]. Here, we systematically examine the distribution of these phosphoinositides on autophagosomal membranes in yeast, *Saccharomyces cerevisiae*, which is a classical model organism of autophagy research.

## 2. Materials and Methods

### 2.1. Strains and Plasmids

Strains used in this work are listed in Supplementary Table S1. Plasmids used in this work, including brief descriptions of the construction process, are listed in Supplementary Table S2. Generally, parental plasmids were treated with the indicated enzymes. Insert fragments were acquired via PCR with the listed primers. DNA fragments were assembled by homologous recombination. Plasmids LDP218 and proATG8-2KatushkasS-Atg8-HYG are previously described [27]. Primers used in plasmids construction and gene knockout are listed in Supplementary Table S3.

BY4741 (wild type) or temperature-sensitive mutants were transformed with linearized plasmids expressing 2Katushka2S-Atg8 and the phosphoinositides probes using the common LiAc method [28]. Transformants with single-copy integration of the plasmids were picked for subsequent analysis.

Point mutations on VPS34 were introduced using the CRISPR/Cas9 method [29]. Briefly, PAM sequences were obtained from E-CRISP (http://www.e-crisp.org/E-CRISP/) (accessed on 14 July 2024) [30] to design the gRNA. Cas9 and gRNA expressing plasmids and mutation templates were co-transformed into yeast cells using the common LiAc method. Correct mutations were then verified by DNA sequencing.

### 2.2. Culturing of Yeast Cells

To culture yeast cells, a single clone was picked from a fresh plate (i.e., storage time of plates at 4 °C less than 2 weeks) and incubated in liquid medium with shaking at 220 rpm. For experiments that did not involve temperature-sensitive mutants, the incubation temperature was 30 °C. For experiments involving temperature-sensitive mutants, the initial incubation temperature was 23 °C, the permissive temperature. Shifting to 37 °C, the non-permissive temperature was used to inactivate the mutants.

For experiments verifying the specificity of phosphoinositides probes, yeast cells were inoculated to SMD + CA medium (6.7% yeast nitrogen base, 5% casamino acid, 20 mg/L methionine, 30 mg/L adenine, 20 mg/L histidine, 20 mg/L methionine, 50 mg/L leucine, 30 mg/L lysine, 50 mg/L tryptophan, 20 mg/L uridine, and 2% D-glucose) and incubated overnight to reach mid-log phase. Care was taken to ensure that yeast cultures never exceeded log-phase during culturing and diluted as necessary during culturing.

For experiments examining autophagy, cells were initially cultured in YPD liquid medium (1% yeast extract, 2% peptone, and 2% D-glucose) to mid-log phase, then shifted to SD-N medium (2% D-glucose, and 0.17% yeast nitrogen base without amino acids and ammonium sulfate) for the appropriate amount of time.

### 2.3. Live Cell Imaging and Analysis

For live cell snapshots, cover glass was coated with 1 mg/mL concanavalin A on one side, to which 125 μL of yeast liquid culture, containing about 10^6^ cells, was loaded and left to precipitate for 5 min at room temperature. The cover glass containing yeast cells was then attached to a glass slide and observed. Image z-stacks were acquired at 0.5 μm stepping, with appropriate channels included. For multi-channel imaging, all channels were acquired before moving to the next z slice. Imaging was performed on an Olympus IX83 inverted microscope with a 100× oil immersion objective (UPLXAPO100XO, Olympus, Tokyo, Japan) and a sCMOS camera (Prime BSI, Photometrics, Huntington Beach, CA, USA).

### 2.4. Data Analysis

The fluorescent puncta and cell numbers were counted manually. Colocalization ratio was calculated by dividing the number of colocalized puncta by the total puncta number of one protein, as indicated on figure labels and legends.

Each experiment was repeated independently for at least three times. Statistical differences were analyzed by one-way ANOVA.

## 3. Results

### 3.1. Confirming the Specificity of the Selected Probes

**Figure 2:**
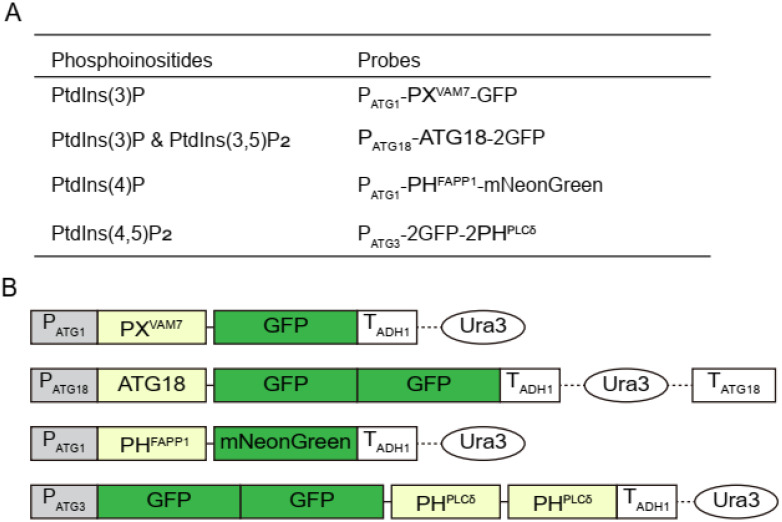
Summary of the phosphoinositide probes used in this study. (**A**) List of phosphoinositides and their probes. For each probe, the promotor used to drive expression, the lipid-binding domain responsible for phosphoinositide recognition, and the fluorescent protein for signal generation are summarized. PX^VAM7^ is the PX domain of yeast Vam7, residue 12–120. ATG18 is the full length of yeast Atg18. PH^FAPP1^ is the PH domain of mouse FAPP1, residue 1–100. 2PH^PLCδ^ is a tandem PH domain of Rat PLCD1, residue 11–140. (**B**) Schematic depiction of phosphoinositide probe constructs. In each probe construct, the gray box represents the promotor; the lime box represents the lipid-binding domain; the green box represents the fluorescent protein; the white box represents terminator, and the oval box represents the selection marker. Black lines between lipid-binding domains and fluorescent proteins indicate domain linkers, dashed line indicates other interlinking sequences.

To visualize the subcellular localization of phosphoinositides in yeast (*Saccharomyces cerevisiae*), we first constructed a set of protein-based probes to specifically label the phosphoinositides in live cells (Figure 2). We used the following domains to construct probes for different phosphoinositides: the PX domain from yeast Vam7 (residue: 12–120) for PtdIns(3)P [30,31], the full-length yeast Atg18 for PtdIns(3)P and PtdIns(3,5)P_2_ [32,33], the PH domain from mouse FAPP1 (residue: 1–100) for PtdIns(4)P [34,35], and the PH domain from rat PLC-δ (residue: 11–140) for PtdIns(4,5)P_2_ [36,37,38]. These domains were fused to GFP or mNeonGreen at either the N- or C-terminus. The constructs were expressed under the control of low-strength promotors, such as P_ATG1_ or P_ATG3_, to avoid saturating the phosphoinositides. We then verified the specificity of these probes by comparing their subcellular distributions in wild-type cells versus those in mutant cells deficient in the production of the corresponding phosphoinositides. Note that to thoroughly survey the subcellular distribution of fluorescent probes, we collected image stacks to cover the entire depth of the cells, i.e., z-stacks. Yeast cells have a typical depth of about 4 μm. In this work, each z-stack consisted of 15 slices that were spaced 0.5 μm apart. For data presentation in subsequent figures, either a representative slice close to the center of the cells (denoted as slice) or a max-intensity projection of the stack (denoted as z-projection) is used.

**Figure 3:**
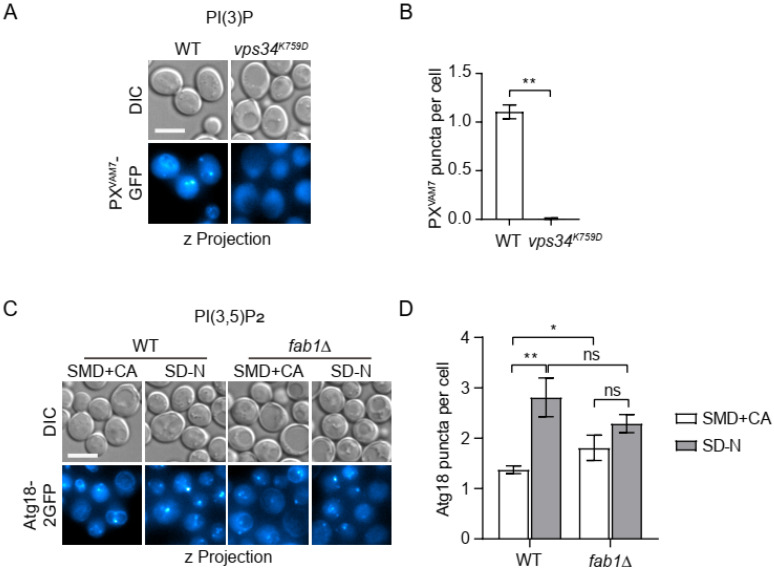
Validating the specificity of PtdIns(3)P and PtdIns(3,5)P2 probes. (**A**,**B**) Specificity of PtdIns(3)P probe, PX^VAM7^-GFP. Wild type (WT) and *vps34^K759D^* cells expressing PX^VAM7^-GFP were cultured in SMD+CA to mid-log phase and imaged by fluorescent microscopy. (**A**) Representative snapshots. (**B**) Numbers of PX^VAM7^-GFP puncta per cell. (**C**,**D**) Specificity of PtdIns(3)P and PtdIns(3,5)P_2_ probe, Atg18-2GFP. WT and *fab1Δ* cells expressing Atg18-2GFP were cultured in SMD + CA to mid-log phase, then shifted to SD-N for 1 h of starvation. Cells were imaged by fluorescent microscopy before and after starvation. (**C**) Representative snapshot. (**D**) Numbers of Atg18-2GFP puncta per cell. Scale bar, 5 μm. Error bar, standard deviation, *n* = 3. **, *p* < 0.01; *, *p* < 0.05; ns, not significant.

In wild-type (WT) cells, our candidate PtdIns(3)P probe, PX^VAM7^-GFP, was present on both vacuolar membrane and cytoplasmic puncta (Figure 3A,B). This pattern was similar to that of GFP-Vam7 [30]. The cellular distribution of GFP-Vam7 was known to depend on its PX domain and the activity of PtdIns(3)K. We then tested whether the subcellular distribution of PX^VAM7^-GFP depended on the PtdIns(3)K, Vps34. In *vps34^K759D^* mutant cells, which carried a point mutation that severely impaired its enzymatic activity [39], the signal of PX^VAM7^-GFP diffused over the cytoplasm (Figure 3A,B). These data support the use of PX^VAM7^-GFP as a PtdIns(3)P probe and demonstrate that PtdIns(3)P is mainly distributed on vacuolar membrane and cytoplasmic punctate structures.

PtdIns(3,5)P_2_ is a relatively low-abundance phosphoinositide in eukaryotic cells [40,41]. Proteins of the WIPI family proteins function as effectors of PtdIns(3)P and PtdIns(3,5)P_2_, many of which are conserved across from yeast to mammals [40,42]. We used the full-length Atg18 as a candidate probe. Atg18-2GFP displayed both vacuolar membrane and cytoplasmic puncta enrichment in wild-type cells. Such a pattern superficially resembles that of the PtdIns(3)P probe, PX^VAM7^-GFP. However, when the biosynthesis of PtdIns(3,5)P_2_ was blocked by deleting the sole kinase [43,44], *FAB1*, the vacuolar membrane localization of Atg18 was significantly reduced, while cytoplasmic puncta were increased (Figure 3C,D). Past studies have demonstrated that WIPI proteins bind PtdIns(3,5)P_2_ and PtdIns(3)P with similar affinity through the same motif [33]. Taking this property into consideration, we conclude that PtdIns(3,5)P_2_ is mainly distributed on the vacuolar membrane and that the cytoplasmic puncta labeled by Atg18-2GFP contains PtdIns(3)P instead.

**Figure 4:**
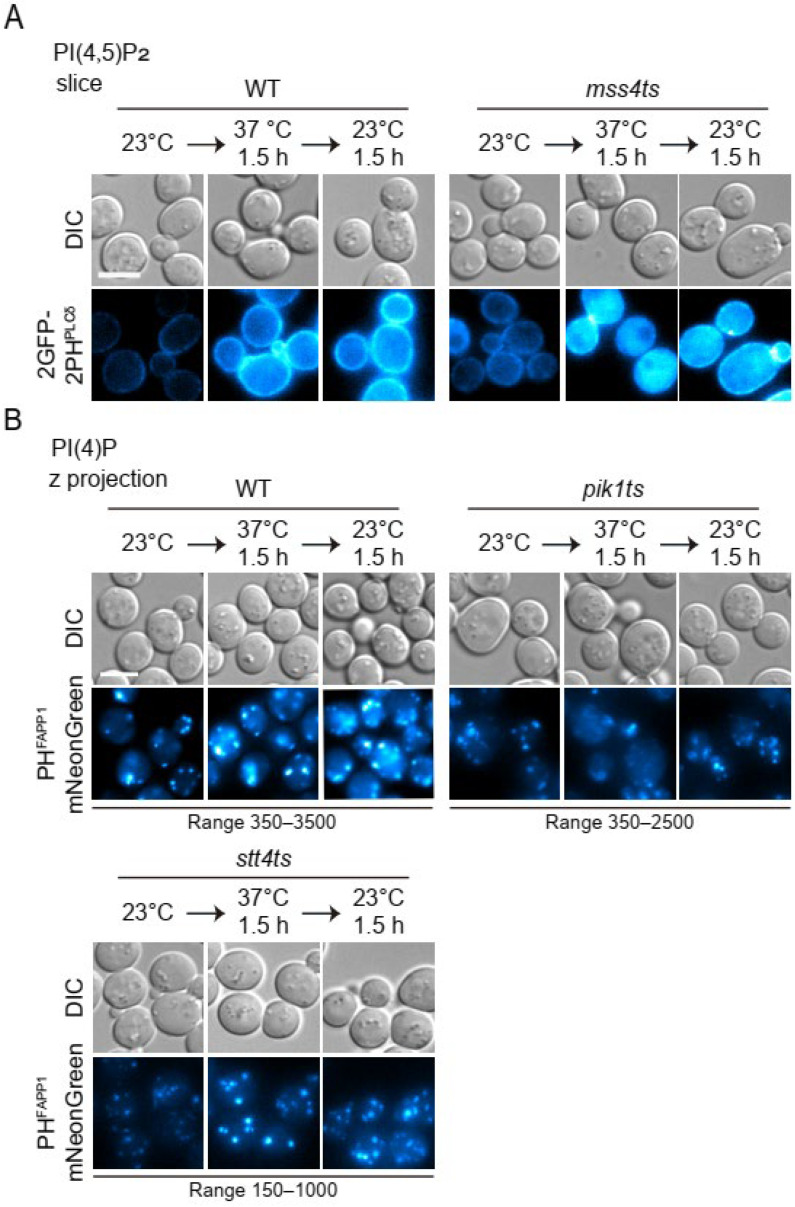
Validating the specificity of PtdIns(4)P and PtdIns(4,5)P_2_ probes. (**A**) Specificity of PtdIns(4,5)P_2_ probe, 2GFP-2PH^PLC-δ^. WT and *mss4^ts^* cells expressing 2GFP-2PH^PLC-δ^ were cultured in SMD + CA initially at the 23 °C permissive temperature to mid-log phase, then subject to temperature shifts as indicated. Cells were imaged before and after the indicated temperature shifts. Representative snapshots are shown. (**B**) Specificity of PtdIns(4)P probe, PH^FAPP1^-mNeonGreen. WT and PtdIns(4)Ks mutants (*pik14^ts^* and *stt4^ts^*) expressing PH^FAPP1^-mNeonGreen were cultured in SMD + CA initially at the 23 °C permissive temperature to mid-log phase, then subject to temperature shifts as indicated. Cells were imaged before and after the indicated temperature shifts. Representative snapshots are shown. Scale bar, 5 μm.

We employed the PH domain of FAPP1 as the candidate PtdIns(4)P probe. FAPP1 localizes to the trans-Golgi network via its PH domain [34,45]. Its PH domain has been widely used for this purpose [34,35,46,47]. In WT cells, PH^FAPP1^-mNeonGreen labeled multiple puncta in the cytosol (Figure 4B). Because the two kinases responsible for PtdIns(4)P production (Pik1 and Stt4) are essential for yeast viability [16], we assessed the specificity of our PH^FAPP1^-mNeonGreen construct in temperature-sensitive conditional mutants of the kinases. As shown in Figure 4B, in *pik1^ts^* mutant cells, PH^FAPP1^-mNeonGreen puncta were significantly reduced at the restrictive temperature of 37 °C; and this reduction was reversible upon shifting back to the permissive temperature of 23 °C. The dynamics of PH^FAPP1^-mNeonGreen in *stt4^ts^* mutant cells were similar to that in WT. These cells displayed an increase in the brightness of PH^FAPP1^-mNeonGreen puncta upon temperature elevation, but not the number. The localization of PH^FAPP1^-mNeonGreen on cytoplasmic puncta, but not on plasma membrane is consistent with previous observations. We conclude that PH^FAPP1^-mNeonGreen is a suitable cytoplasmic PtdIns(4)P probe.

The PH domain of PLC-δ exhibited high binding specificity and affinity to PtdIns(4,5)P_2_ in vitro and in vivo [36,37]. We used tandem copies of this domain to construct our PtdIns(4,5)P_2_ probe. In yeast, PtdIns(4,5)P_2_ was produced from PtdIns(4)P via Mss4, which is essential for viability. In both WT and *mss4^ts^* mutant cells, 2GFP-2PH ^PLC-δ^ exhibited plasma membrane distribution at permissive temperature (Figure 4A). Upon inactivation of *mss4* at the restrictive temperature, 2GFP-2PH^PLC-δ^ redistributed from the plasma membrane to the cytosol (Figure 4A). The change could be reversed by shifting the temperature back. These data indicate that 2GFP-2PH^PLC-δ^ is a suitable probe for PtdIns(4,5)P_2_.

### 3.2. Probing the Distribution of Phosphoinositides on Autophagosomal Membrane

**Figure 5:**
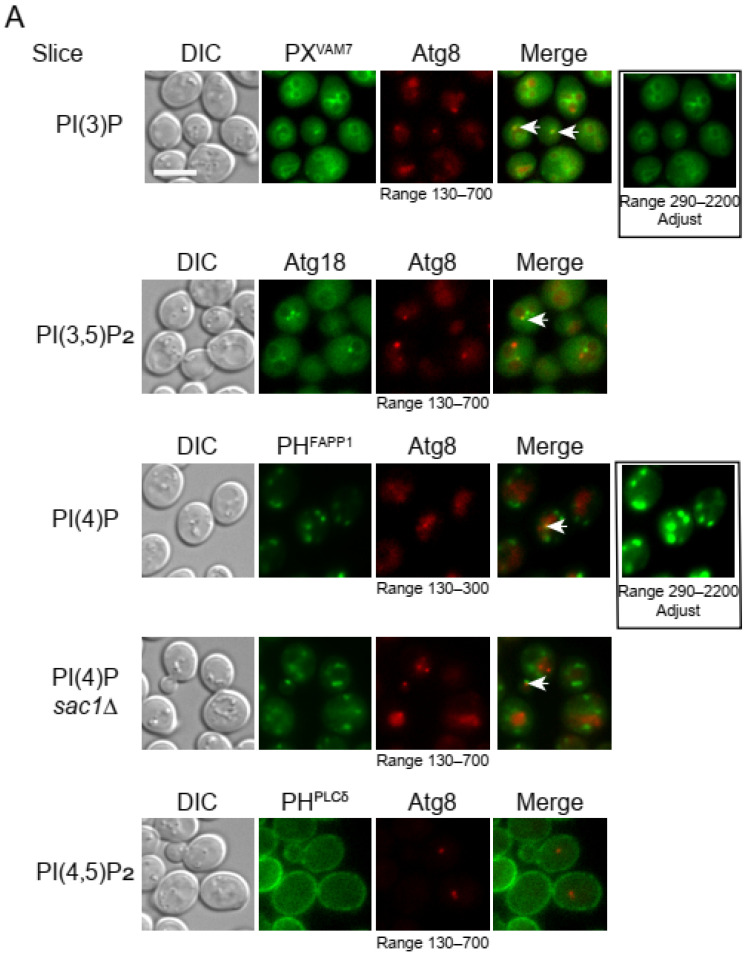

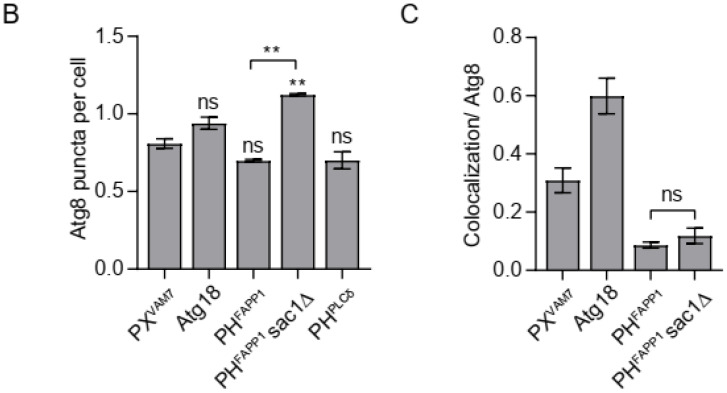
Evaluating the co-localization of phosphoinositides probes with an autophagosomal membrane marker, 2Katushka2S-Atg8. (**A**) Representative snapshots of WT yeast cells co-expressing a phosphoinositide probe and 2Katushka2S-Atg8 under starvation. Cells were cultured in SMD + CA to mid-log phase, then shifted to SD-N for 1 h of starvation and imaged. Black box to the right, green channel images adjusted to display the same intensity range. Arrows indicate colocalized puncta. (**B**) Atg8 puncta per cell. (**C**) Ratios of Atg8 puncta positive of a co-expressed phosphoinositide probe. The ratio was calculated as the number of colocalized puncta/total number of Atg8 puncta. Scale bar, 5 μm. Error bar, standard deviation, *n* = 3. **, *p* < 0.01; ns, not significant.

Subsequently, we investigated the potential presence of phosphoinositides on autophagosomal membranes using the aforementioned probes. We used 2Katushka2S-Atg8 (Katushka2S is a red fluorescent protein) to label the autophagosomal membrane. During autophagosome formation, Atg8 is covalently conjugated to a membrane lipid, phosphatidylethanolamine (PE), leading to its association with the autophagosomal membrane [48]. Yeast cells were incubated in a nitrogen starvation medium for 1 h to induce autophagy. Under such conditions, the overall distribution patterns of our phosphoinositides probes are similar to those under nutrient-rich conditions. Furthermore, co-expressing of these probes did not substantially affect Atg8 puncta formation (Figure 5A,B).

Among the four phosphoinositides probes tested, we observed substantial co-localization between 2Katushka2S-Atg8 and two probes: PtdIns(3)P probe PX^VAM7^-GFP, and PtdIns(3)P/ PtdIns(3,5)P_2_ dual specificity probe Atg18-2GFP (Figure 5A,C). We verified that under starvation conditions, the deletion of *FAB1* changed the distribution of Atg18-GFP from one consisting of vacuolar membrane and cytoplasmic puncta to one consisting primarily of cytoplasmic puncta (Figure 3C,D). This is similar to what we observed for yeast cells under nutrient-rich conditions (Figure 3C,D). Taken together, these data indicate that under nitrogen starvation, PtdIns(3)P is present in substantial amounts at the site of autophagosome formation and that PtdIns(3,5)P_2_ is primarily located on the vacuolar membrane.

We only observed occasional co-localization between 2Katushka2S-Atg8 and PtdIns(4)P probe, PH^FAPP1^-mNeonGreen (Figure 5A,C). When the phosphatase responsible for PtdIns(4)P hydrolysis, *SAC1*, was deleted, we observed a moderate increase in the number of Atg8 puncta, but the ratios of colocalization with PH^FAPP1^-mNeonGreen remained low at about 0.1. The low co-localization rates were not the result of a low fluorescent signal. In fact, the brightness of PH^FAPP1^-mNeonGreen was higher than that of PX^VAM7^-GFP (Figure 5A, block boxes). At such a low rate of co-localization, we suspect that some of the observed cases of colocalization represent random passage of PtdIns(4)P-containing structures near Atg8 puncta.

For the PtdIns(4,5)P_2_ probe, 2GFP-2PH^PLC-δ^, we did not see any punctate signal co-localizing with Atg8. Its signal remained primarily on the plasma membrane under starvation conditions (Figure 5A). This corroborated a previous report that Mss4 was not involved in the regulation of autophagy in yeast [19].

## 4. Discussion

In this study, we systematically investigated the distribution of several phosphoinositides on autophagosomes in the yeast model organism. We utilized fluorescent protein-tagged probes to label different phosphoinositides, and 2Katushka2S-Atg8 as a marker for autophagosomal membrane. The dependence on the corresponding kinases for the association of our phosphoinositide probes to specific subcellular sites suggests that they are valid phosphoinositide probes. Our live cell imaging found no evidence for the presence of PtdIns(4,5)P_2_ and PtdIns(3,5)P_2_ at the site of autophagosome formation. PtdIns(4,5)P_2_ and PtdIns(3,5)P_2_ appeared to concentrate at the plasma membrane and vacuolar membrane. Our data indicate that PtdIns(3)P is the most abundant phosphoinositide present at the site of autophagosome formation. Despite PtdIns(4)P being the most abundant phosphoinositide in the cell [7,41], it is not present at the site of autophagosome formation in substantial amounts.

Our visualization of PtdIns(3)P probes at the site of autophagosome formation is consistent with the current understanding in the autophagy field that PtdIns(3)P is locally produced in the early stage of autophagosome formation [14]. This pool of PtdIns(3)P is produced by a PtdIns(3)K complex containing Vps34, Vps15, Vps30/Atg6, Atg14, and Atg38 [49,50,51]. The pool of PtdIns(3)P is later hydrolyzed, which explains why the colocalization ratio is lower than 1 [52]. PtdIns(3)P recruits important downstream effectors, including a lipid transfer complex containing Atg2 and Atg18, and a sorting nexin complex containing Atg24 [53,54]. These complexes play important roles in the expansion and morphogenesis of the autophagosomal membrane.

Our finding of the absence of PtdIns(4,5)P_2_ at the site of autophagosome formation agrees with previous research showing that Mss4, the PtdIns(4)P 5-kinase, is not involved in the regulation of autophagic flux [19]. On the other hand, PtdIns(4,5)P_2_ has been shown to regulate autophagy in animal cells [15,20,21,22]. Importantly, in animal cells, PtdIns(4,5)P_2_ at the site of autophagosome formation is produced by PtdIns(5)P 4-kinases using PtdIns(5)P as the substrate. PtdIns(5)P 4-kinase is not present in yeast, suggesting that this part of the regulatory network is subject to re-wiring in evolution [21].

PtdIns(4)P, the most abundant phosphoinositides in eukaryotic cells, plays a crucial but still not fully understood role in autophagy regulation [7,41]. The inactivation of either kinases (pik1 or stt4) or phosphatases (sac1) almost completely blocks the autophagic flux in yeast [19,26]. However, our live cell imaging suggests that at the site of autophagosome formation, PtdIns(4)P is either transiently present at a very low amount, or not present at all. In the latter scenario, PtdIns(4)P may regulate autophagy indirectly by controlling Atg9 trafficking. Atg9 is a transmembrane protein with lipid scramblase activity [19,55]. Atg9 is crucial for the initiation of autophagosome formation. In yeast, when autophagy is induced under restrictive temperatures, the anterograde trafficking of Atg9 is impaired in *pik1^ts^* cells [19]. Atg9 vesicles are known to bud from the late Golgi during yeast autophagy, suggesting that PtdIns(4)P may regulate autophagy via the Golgi apparatus. An alternative model has been proposed from research in animal cells [55]. ARFIP2, a trans-Golgi protein, has been found to be enriched on isolated ATG9A vesicles. This protein can recruit PI4KIIβ (the yeast homolog of which is Lsb6) to ATG9A vesicles, forming a complex with ULK1 that facilitates the initiation of autophagy. However, it remains to be determined whether the PtdIns(4)P produced by PI4KIIβ on ATG9A vesicles directly participates in this process. Different models have been proposed to explain the significance of PtdIns(4)P hydrolysis in autophagy. One possibility is that the excess PtdIns(4)P on the autophagosome could hinder the recruitment of certain SNAREs, such as Vam7 [25,26], which are necessary for fusion with the lytic compartment. The other one is that the excess PtdIns(4)P on the autophagosome may promote aberrant interactions between autophagosome and ER via OSBP-mediated contact site formation [56], thereby disrupting the fusion of the autophagosome with lysosomes. Under our experimental condition, we did not see a substantial presence of PtdIns(4)P in *sac1Δ* mutant cells. Although at this stage we could not rule out the possibility that PtdIns(4)P may accumulate to high levels under more extreme conditions to trigger similar inhibitory effects, we suspect that the role of PtdIns(4)P hydrolysis in yeast autophagy may lie in other yet to be discovered steps. More work is needed to elucidate the roles of PtdIns(4)P production and hydrolysis in autophagy.

## Data Availability

The original contributions presented in the study are included in the article/Supplementary Materials, further inquiries can be directed to the corresponding authors.

## Supplementary Materials

The following supporting information can be downloaded at: https://www.mdpi.com/article/10.3390/microorganisms12071458/s1, Table S1: Strains; Table S2: Plasmids; Table S3: Primers.
