# Supplementary material for: Assessing the Presence of Phosphoinositides on Autophagosomal Membrane in Yeast by Live Cell Imaging
Source: Microorganisms. 2024 Jul 18;12(7):1458. doi: 10.3390/microorganisms12071458 (PMC11279164; doi:10.3390/microorganisms12071458)
Supplement: Supplementary file 1 [file microorganisms-12-01458-s001.zip › Table S1 yeast strains-202400711.pdf]

Table S1 Strains

Name	Genotype
FS1	BY4741 T_{ADH1}:: P_{ATG1}-PX(Vam7$^{12\text{-}120}$)- GFP-Ura3
FS2	BY4741 *vps34*::*vps34*^{K759D} T_{ADH1}:: P_{ATG1}-PX(Vam7$^{12\text{-}120}$)- GFP -Ura3
FS3	BY4741 ATG18:: 2GFP-Ura3
FS4	BY4741 *fab1*Δ:: KanMX6 ATG18:: 2GFP-Ura3
FS5	BY4741 P_{ATG3}:: P_{ATG3}-2GFP-2PH(PLC-δ$^{1056\text{-}1176}$)-Ura3
FS6	BY4741 *mss4*^{ts} P_{ATG3}:: P_{ATG3}-2GFP-2PH(PLC-δ$^{1056\text{-}1176}$)-Ura3
FS7	BY4741 T_{ADH1}:: P_{ATG1}-PH(FAPP1$^{1\text{-}100}$)-mNeonGreen-Ura3
FS8	BY4741 *stt4*^{ts} T_{ADH1}:: P_{ATG1}-PH(FAPP1$^{1\text{-}100}$)-mNeonGreen-Ura3
FS8	BY4741 *pil1-139* T_{ADH1}:: P_{ATG1}-PH(FAPP1$^{1\text{-}100}$)-mNeonGreen-Ura3
FS9	BY4741 T_{ADH1}:: P_{ATG1}-PX(Vam7$^{12\text{-}120}$)- GFP -Ura3 ATG8:: P_{ATG8}-2Katushka2S-ATG8-HYG
FS10	BY4741 ATG18:: 2GFP-Ura3 ATG8:: P_{ATG8}-2Katushka2S-ATG8-HYG
FS11	BY4741 T_{ADH1}:: P_{ATG1}-PH(FAPP1$^{1\text{-}100}$)-mNeonGreen-Ura3 ATG8:: P_{ATG8}-2Katushka2S-ATG8-HYG
FS12	BY4741 P_{ATG3}:: P_{ATG3}-2GFP-2PH(PLC-δ$^{1056\text{-}1176}$)-Ura3 ATG8:: P_{ATG8}-2Katushka2S-ATG8-HYG
FS13	BY4741 T_{ADH1}:: P_{ATG1}-PH(FAPP1$^{1\text{-}100}$)-mNeonGreen-Ura3 ATG8:: P_{ATG8}-2Katushka2S-ATG8-HYG
