# Supplementary material for: Assessing the Presence of Phosphoinositides on Autophagosomal Membrane in Yeast by Live Cell Imaging
Source: Microorganisms. 2024 Jul 18;12(7):1458. doi: 10.3390/microorganisms12071458 (PMC11279164; doi:10.3390/microorganisms12071458)
Supplement: Supplementary file 1 [file microorganisms-12-01458-s001.zip › Table S2 Plasmids-20240712.pdf]

Table S2 Plasmids

Plasmid name	Parental plasmid	Restriction sites	Primers for insert amplification
proATG1-PX(Vam7$^{12-120}$)-GFP-Ura	ClhN-GFP-Ura	EcoRI	pAtg1-F/pAtg1-R, Vam7-PX-F/Vam7-PX-R
proATG1-PH(FAPP1$^{1-100}$)-mNeonGreen-Ura	ClhN-mNeonGreen-Ura	EcoRI	pAtg1-F/pAtg1-R, Fapp1-PH-F/Fapp1-PH-R
proATG3-2GFP-2PH(PLC-δ$^{11-140}$) -RS406	RS406	SnaBI	pAtg3-F/pAtg3-R, GFP1-F/GFP1-R, GFP2-F/GFP2-R, Avo1-PH-1-F/Avo1-PH-1-R, Avo1-PH-2-F/Avo1-PH-2-R
