# Supplementary material for: Assessing the Presence of Phosphoinositides on Autophagosomal Membrane in Yeast by Live Cell Imaging
Source: Microorganisms. 2024 Jul 18;12(7):1458. doi: 10.3390/microorganisms12071458 (PMC11279164; doi:10.3390/microorganisms12071458)
Supplement: Supplementary file 1 [file microorganisms-12-01458-s001.zip › Table S3 primers.pdf]

Table S3 Primers

Name	Sequence 5'-3'
pAtg3-F	CCTTCGGTGGGTCCAGACC
pAtg3-R	ATGATAATAAAATTGTATTTACTCCTTTGTACTTCTTTG
GFP1-F	TTAGGAACAAAGAAGTACAAAGGAGTAAATACAATTTTATTATCATC GATATGTCTAAAGGTGAAGAATTATTCACTGGT
GFP1-R	GAATGGCTTCTCAAGATCACGCGTTGGAACTGGAATTTTGTACAATTC ATCCATACCATGGGTAAT
GFP2-F	ATTCCAGTTCCAACGCGTGATCTTGAGAAGCCATTCATGTCTAAAGGT GAAGAATTATTCACTGGT
GFP2-R	CCGTGGGATCTGAGTCCGGAACCACCACCAGAACCGCCTCCAGAACC GCCTCCACTAGTTTTGTACAATTCATCCATACCATGGGTAATA
pAtg1-F	ACCTGCCACAAGGTTATTTCTACAC
pAtg1-R	TTTCTTAATTTCTCGTCTGGTGTTGTAAAA
Vam7-PX-F	ATATTTTCAAATCTCTTTTACAACACCAGACGAGAAATTAAGAAAAT GTTAAGAATCAAGGTGGACGATGTTAAAATCAA
Vam7-PX-R	CAACTGCAGGAAGTCTTGCGCTA
Fapp1-PH-F	CAACACCAGACGAGAAATTAAGAAAATGGAGGGGGTGTTGTACAAG TG
Fapp1-PH-R	AGTCCTTGTATCAGTCAAACATGCTTTG
Avo1-PH-1-F	TCTGGTGGTGGTTCCGGACTCAGATCCCACGGGCTCCAGGATGACC
Avo1-PH-1-R	GCGGATTCCGCAAGGGCGAATTCGCTGCGGACGGCCGCTGCCTTCTG CCGCTGGTCCATG
Avo1-PH-2-F	GCAGCGAATTCGCCCTCGGGGAATTCGCCGCGGCAGCTCACGGGCTC CAGGATGACC
Avo1-PH-2-R	TATTTAGAAGTGGCGCGCCTCACCTAGGCTACTTCTGCCGCTGGTCCA TG
Sac-D1	TAACGATAATATTTATATACACGTATATTTTCTCGTCTAGAT GCTTCGTACGCTGCAGGTCG
Sac1-LRS	TTTTGGATTTACAATAATCATCATTTTATCACATATAGAACTCATTA GCATAGGCCACTAGTGGATC
